# Design and characterization of sustainable mortars incorporating industrial waste–derived materials

**DOI:** 10.1038/s41598-026-41743-5

**Published:** 2026-03-04

**Authors:** Mihaela Caftanachi, Mihai Vrabie, Maria Harja, Roxana Dana Bucur, Daniel Bucur

**Affiliations:** 1 Faculty of Chemical Engineering and Environmental Protection, Gheorghe Asachi Technical University of Iasi, 73 Mangeron Boulevard, Iasi, Romania; 2 Department of Control, Expertise and Services, Faculty of Food and Animal Sciences, “Ion Ionescu de la Brad” Iasi University of Life Sciences, 8, Mihail Sadoveanu Alley, 700490 Iasi, Romania; 3 Department of Pedotechnics, Faculty of Agriculture, , “Ion Ionescu de la Brad” Iasi University of Life Sciences, 3, Mihail Sadoveanu Alley, 700490 Iasi, Romania

**Keywords:** Sustainable mortar, Fly ash, Slag, Resource valorisation, mechanical properties, Engineering, Environmental sciences, Materials science

## Abstract

Environmental pressures necessitate finding alternatives to Portland cement using alkali-activated aluminosilicate materials. Mortars containing different types of class F fly ash (Romania and Canada), furnace slag, silica fume and sand, activated with 5.1 M potassium hydroxide solution and 40% sodium silicate solution. Three mixes were tested (Mix 1 – fly ash Romania, Mix 2 - fly ash Canada and Mix 3 – fly ash Canada and slag). The activators were chosen to develop materials with high mechanical strength. The activation process occurred at controlled laboratory conditions (20 ± 2℃ and 65% relative humidity). Mix 3 showed the highest strength values of 43.8 MPa and a flexural strength of 7.4 MPa after 28 days. Mix 2 had a compressive strength of 26.0 MPa, in contrast to 8.3 MPa for Mix 1. The addition of blast furnace slag (Mix 3 vs. Mix 2) nearly doubled the compressive strength values. Proposed mixes are an effective alternative to cement mortar that reduce waste, through valorization of industrial by-products and diminish carbon dioxide emissions by 30%. Microstructural analyses (SEM and XRD) confirmed the formation of reaction products consistent with the observed mechanical strength. The developed mixes show promising mechanical performance and contribute to reducing CO₂ emissions in construction materials.

## Introduction

Currently, global warming is a significant problem for the whole world. In certain industries, human activity has been the main cause of warming. Thus, many countries, both developing and developed, are now striving to use cleaner and less polluting technologies to manufacture building materials^1,3^. The release of greenhouse gas emissions like carbon dioxide (CO_2_) into the atmosphere through industrial activities and transportation is the primary driver of global warming. About 65% of the greenhouse gas emission contribution to global warming comes from CO_2_^4,5^.

The rapid development of infrastructure has resulted in an increased need for Portland cement (OPC), the main component of construction materials. Global cement production represents up to 7% of CO_2_ emissions, along with a significant reduction of natural resources. Currently, approximately 0.87 tons of greenhouse gas emissions are released per ton of cement^6^. The cement industry consumes a large amount of energy and is responsible for the production of considerable amounts of CO_2_ emissions because the production of cement clinker takes place at high temperatures (1450℃)^7^.

Consequently, to protect the environment, the construction industry must find alternatives to OPC. Based on existing studies, an alternative material could be one that uses alkali-activated materials, without including cement in its composition^8^. Alkali-activated materials have attracted the attention of researchers for their development due to excellent and promising properties such as superior values for compressive and flexural strength^9^, low permeability, corrosion prevention in an acidic environment as well as good resistance to freeze–thaw cycles^10^. These alkali materials do not require elevated temperatures for calcination or sintering and are also more easily obtained than those materials based on OPC. As a result, the reaction driving these new materials can occur at room temperature, leading to the production of high-quality materials suitable for use as eco-friendly construction materials with excellent mechanical and chemical properties^11,12^. Alkali-activated materials emit a small amount of CO_2_, with a negligible presence of NO_x_, SO_x_ or CO^13^, are resistant to high temperatures, and reduce toxic waste in the environment^14^. Its structure is influenced by the nature and characteristics of the raw material. Sources of aluminosilicates can be metakaolin, various ashes (from power plants, incineration, rice husk ash), granulated blast furnace slag, red mud, and phosphogypsum, among others^15^. In the present study, fly ash, slag, and silica fume were used.

The recovery of industrial by-products benefits environmental protection because it can help overcome the limited storage capacity and the unmanaged disposal of by-products in landfills. Guo et al.^16^ offered three solutions to reduce CO_2_ emissions from the cement industry: reducing the amount of limestone in cement, reducing the amount of cement in concrete, and reducing the number of constructions that use cement. Additionally, Mehta^17^ suggested two solutions for producing environmentally sustainable concrete. The first, a short-term initiative called ‘industrial ecology’, focuses on minimising the use of natural resources, reducing energy consumption, and lowering CO_2_ emissions. Reducing the impact of unwanted industrial by-products is the long-term vision, and this can be achieved by reducing the rate of material consumption.

Aluminosilicate sources have different physical, chemical and mineralogical characteristics^18^. Despite the extensive research on alkali-activated materials, most reported studies use relatively high-molarity alkaline solutions (typically 5–12 M) and elevated curing temperatures ranging from 45 to 80 °C to achieve satisfactory mechanical performance. In addition, while mechanical properties are frequently investigated, the combined assessment of low-molarity activation under ambient curing conditions together with a comparative evaluation of CO₂ emissions relative to OPC systems remains insufficiently explored. Approaches based on high molarity alkaline solutions and elevated curing temperatures involve additional energy consumption, higher operational costs and more restrictive handling conditions, which limit their applicability on an industrial scale. In addition, the need for heat treatments and high alkaline concentrations raises issues related to safety, equipment durability and environmental impact associated with the production of activators. These constraints reduce the feasibility of large-scale implementation, especially in contexts where truly sustainable and economically competitive solutions compared to OPC are targeted. For these reasons, there is a need for systematic investigation of alkali-activated mortars synthesized under milder activation conditions, using readily available industrial by-products, in order to evaluate both their structural performance and environmental impact. Therefore, the production of alkali materials is influenced by various parameters^19,20^, such as the hardening method, alkali concentration, mixture ratios and properties of the materials used^21^. The effects of major factors on obtaining new materials depend on the type of chemical activator, the size distribution of material particles, and the methods of production. The materials developed using alkali activation are called geopolymers or alkali-activated materials. The term geopolymer was named by Joseph Davidovits in 1972^22^. Although geopolymers are classified as zeolites in the aluminosilicate family, they are essentially amorphous polymers^23^. The main difference between geopolymers and alkali-activated materials lies in their chemical composition and reaction mechanisms. The geopolymers are formed through polycondensation of aluminosilicate materials (e.g., metakaolin, fly ash) in an alkaline environment, creating a three-dimensional polymeric network with Si-O-Al bonds. Alkali-activated materials include both geopolymers and alkali-activated cementitious systems. Their obtaining reaction involves dissolution, gelation, and precipitation rather than polymerisation. Geopolymers consist of an amorphous to semi-crystalline aluminosilicate network, similar to synthetic zeolites, while alkali-activated materials may develop C-A-S-H (calcium–alumino–silicate–hydrate) gels, similar to OPC hydration products. The mechanical performance of alkali-activated materials is strongly governed by the structure and continuity of the amorphous to semi-crystalline aluminosilicate network formed during activation. All geopolymers are alkali-activated materials, but not all alkali-activated materials are geopolymers.

Alkali material is synthesised by mixing reactive aluminosilicate materials (i.e., materials containing more or less CaO) with concentrated NaOH or KOH solutions and then curing at different temperatures^24^. By mixing with an alkaline solution, the aluminosilicate sources are dissolved to form tetrahedral units of SiO_4_ and AlO_4_^25^. From a materials science perspective, alkali-activated binders can be regarded as condensed solid systems in which the spatial organisation of aluminosilicate units directly influences the macroscopic behaviour. As the reaction progresses, water is separated out and these tetrahedral units are alternately linked to produce polymeric precursors (–SiO_4_–AlO_4_–; –SiO_4_–AlO_4_–SiO_4_–; –SiO_4_–AlO_4_–SiO_4_–AlO_4_–) by connecting the oxygen atoms into tetrahedral units, resulting in the formation of monolithic alkali material products^26,27^.

Thermogravimetry offers valuable information about the success of activation. In order to determine the thermal stability of the alkali-activated materials and their oxidation/decomposition processes, thermogravimetric analysis can be performed in an oxidising atmosphere (air). The mechanism and number of mass loss stages provide valuable information regarding the stability of the aluminosilicate gel formed during activation. In the literature, two-stage mass loss patterns have been observed in alkali-activated materials. The first mass loss event suggests that the gel retains a certain amount of bound water, which is typical of well-formed gels that are not overly dehydrated or prone to premature breakdown. The second mass loss, occurring at higher temperatures, reflects the degree of stability of the gel network. Recent studies^28^ have shown that the thermal stability of the aluminosilicate gel improves with increased curing temperature or the use of specific activators, such as sodium silicate or sodium hydroxide, which can delay the onset of significant mass loss at higher temperatures.

Therefore, the use of alkali-activated technology contributes to the protection of the environment through the use of pozzolanic industrial by-products, easily procured globally, while the cost–benefit ratio for the use of industrial waste is exceptional compared to the traditional cement products used in industry today. As advantages, we can also mention the fact that these materials can be obtained at ambient temperature and with low CO_2_ emissions^29^.

The scalability of alkali-activated materials depends on the availability of raw materials in consistent quality at large volumes and the efficiency of production processes. Recently, the environmental benefits of alkali-activated materials have been recognised, leading to the development of standards and frameworks aimed at encouraging their use in construction. For instance, the European Union promotes sustainable construction materials, including alkali-activated materials, through the implementation of the European Union’s Green Deal and its Carbon Neutrality agenda. Several countries, including the UK, Germany, and the Netherlands, have introduced regulations that allow for the use of alkali-activated materials as alternatives to traditional cement, provided they meet specific performance and durability criteria. Moreover, ongoing research and development are focused on improving the performance and cost-effectiveness of alkali-activated materials, as well as making the production process more efficient^30^.

This study’s objective is to synthesize and characterize alkali-activated materials using two class F ashes (Canadian and Romanian), silica fume, and furnace slag for usage as OPC mortar cement replacement. To the best of our knowledge, the formulations proposed in this study have not been previously reported, but a key aspect of the study is the investigated activation solution with a maximum of 5.1 M KOH and curing at ambient temperature. In these mild conditions, the obtained materials exhibited superior mechanical properties compared with OPC mortar. Another objective, was studying CO₂ emissions for AIMs production and comparing it with OPC mortar production. Based on the obtained results, this study demonstrates a unique combination of materials and processing conditions, indicating that alkali-activated mortars synthesized at room temperature using a low-molarity (3.8 or 5.1 M) potassium hydroxide activator and readily available industrial by-products can simultaneously achieve the required mechanical performance and considerable CO₂ reduction, positioning them as an effective alternative to OPC mortars.

## Materials and methods

This study used the following: fly ash (FA) class F from CET Iasi, Romania; FA class F from Canada; undensified silica fume from HSH Chemie; and furnace slag from Galati, Romania. Quartz sand, with different grain sizes, supplied by S.C. Bega Minerale Industriale SA (38% with a particle size between 0 and 1 mm, 26% with a particle size between 0 and 0.3 mm, and 36% with a particle size between 0 and 0.5 mm).

The activation was realised with 5.1 M potassium hydroxide (KOH) solution and sodium silicate solution (supplied by PQ Corporation, The Netherlands) that contained 13.6% Na_2_O and 30.9%. SiO_2_. The KOH solution was prepared using KOH flakes of 98% purity and distilled water. All materials were used without any processing.

The characteristics and properties of alkaline inorganic materials (AIMs) depend on many factors, so it is necessary to determine the optimal parameters specific to the raw material, the alkali activation solution, the drying step and the mixing step. The main parameter for obtaining AIMs is the aluminosilicate source. Wastes have specific chemical compositions, with different oxide content, particle size and geometry, which change the properties of the inorganic material.

Therefore, microstructural characterisation is essential to understand the relationship between phase development, internal structure and the resulting mechanical properties of these materials.

The raw materials were subjected to scanning electron microscopy (SEM; SEM JEOL JSM 6390), X-ray diffraction (XRD; X’Pert PRO MRD, PANalytical, having 2θ range between 0 and 90) and ener-gy-dispersive X-ray spectroscopy (EDX) analysis for characterisation. The final products were tested for their mechanical strength, microstructural properties and mass losses at high temperatures. Mass losses were determined using a Mettler Toledo TGA-SDTA851e instrument that recorded simultaneous thermo-gravimetric, derived thermogravimetric and differential thermal analysis curves, the analysis was in the range 20 to 900 °C, with heating rate of 10 °C/min.

### Sample preparation

Based on previous experimental work and the relevant scientific literature^8,9,12,19^, were chosen AIMs binder compositions (Table 1). The mixtures prepared and analysed were: Mix 1 (FA Romanian), Mix 2 (FA Canada) and Mix 3 (FA Canada and slag). In all the mixtures, different additions of silica fume (4%) and 70–72 (%) sand were used.

**Table 1:** The composition of binders (m^3^).

Compounds	FA	Slag	Silica Fume	Sand
Control sample	288 kg of OPC - CEM II/A-LL 42.5 R			720
Mix 1	240	-	40	720
Mix 2	240	-	40	720
Mix 3	160	100	40	700

Synthesis of the new material took place in three stages: solid–solid mixing, liquid–liquid mixing and solid–liquid mixing. Firstly, the solid constituents (binders compounds presented in Table 1) were measured and mechanically mixed, with a mechanical paddle type mixer. The obtained mixtures were then combined with the liquid phase (activator), which had been prepared using KOH solution and sodium silicate solution (cooled at 20℃). To achieve the desired consistency and, most importantly, to ensure good homogenisation, for all samples the mixing was carried out for 10 min. The temperature and relative humidity were always noted, as well as the time when the mixing was completed, and greater attention was required for the period of working with the material. The powdery part was dry to control the water content of the mortars.

To establish the influence of hydroxide concentration, Mix 2 was prepared with 3.8 M KOH and 5.1 M KOH solutions (for an alkaline solution/binder ratio of 0.42). The effect of the alkaline solution/binder ratio was tested considering Mix 2 activated with KOH 5.1 M at ratios of 0.35, 0.37, 0.42, and 0.48. The alkaline solution/binder ratio was selected to achieve plastic consistency based on the flow table test, resulting in a diameter spread of 140 to 180 mm in accordance with SR EN 13395-1:2003. The mechanical properties were tested for a ratio of 0.42, with a diameter spread of 160 mm.

**Fig. 1:**
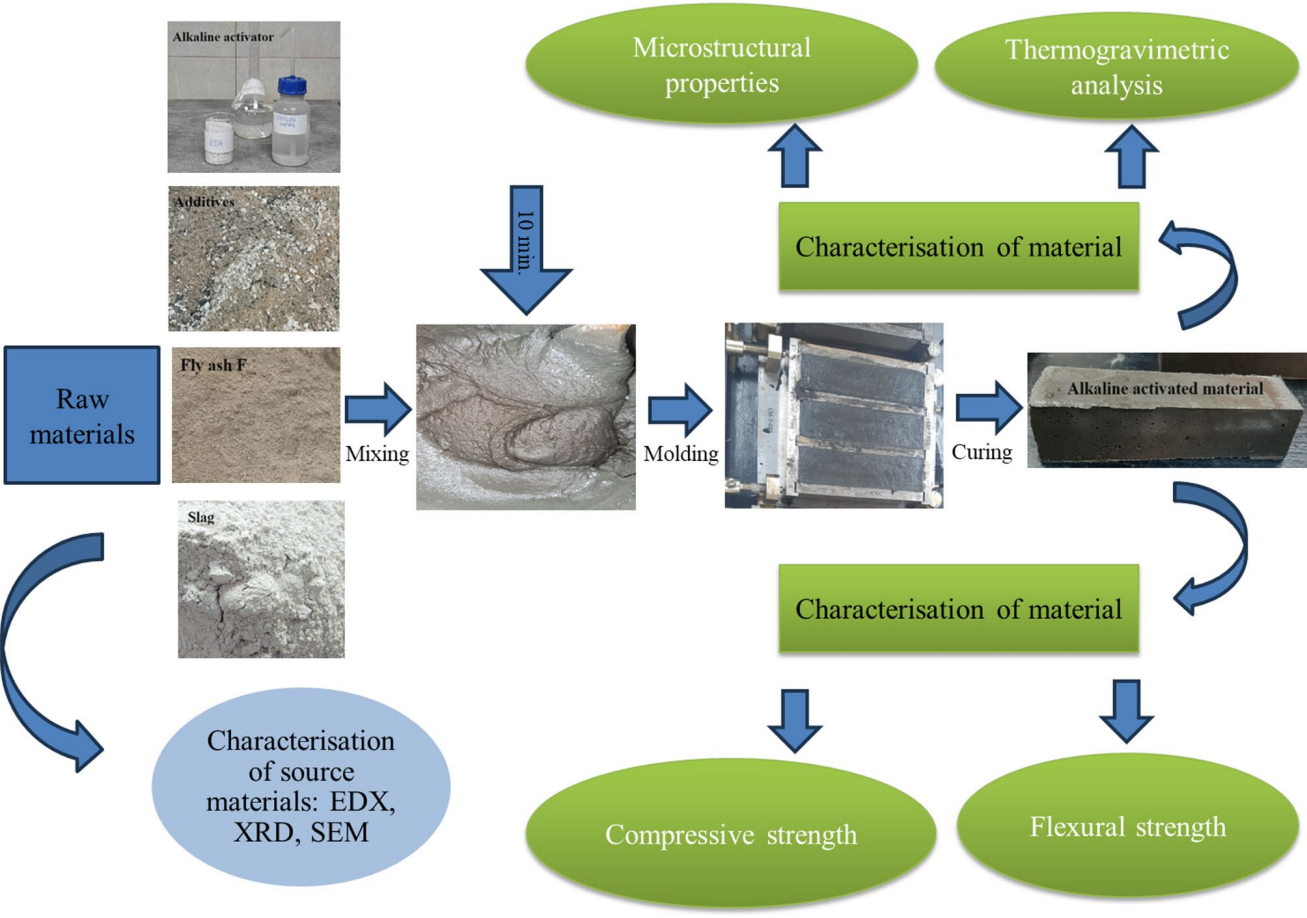
Synthesis of new alkali-activated materials.

The fresh AIMs were poured into moulds and compacted with the vibrating table. The compaction process guarantees uniform compaction conditions, enhances the reproducibility of test specimens, and reduces variability in mechanical qualities. The materials obtained were wet-cured by sealing the moulds with polyethylene film for at least 24 h, after which the samples were demoulded, labelled, and air-cured at approximately 20 ± 2℃ until being subjected to the mechanical tests, in accordance with SR EN 12190:2002 (compressive strength) and SR EN 1015-11:2020 (flexural strength). A 15/250kN hydraulic press was used to perform the flexural and compression tests. The prismatic samples with dimensions of 40 × 40 × 160 and cubic samples with dimensions of 40 × 40 × 40 mm were used. To confirm the reproducibility of the measurements, five parallel measurements were conducted. The AIMs were kept in a laboratory at room temperature (20 ± 2℃) and 65% relative humidity. Figure 1 shows the procedure for obtaining the inorganic materials using alkali activation.

The control sample was prepared using OPC - CEM II/A-LL 42.5 R type, supplied by Heidelberg Materials România S.A. CEM II/A-LL 42.5 R is a Portland-composite cement containing over 80% clinker and less than 20% limestone. It was prepared with a cement/sand ratio of 1:2.5, a water/cement ratio of 0.5, and 0.8% superplasticizer (sodium naphthalene sulfonate) relative to the mass of the binder; the sand was the same type as for AIM materials.

## Results and discussion

### Materials characterisation

#### Fly ash

**Fig. 2:**
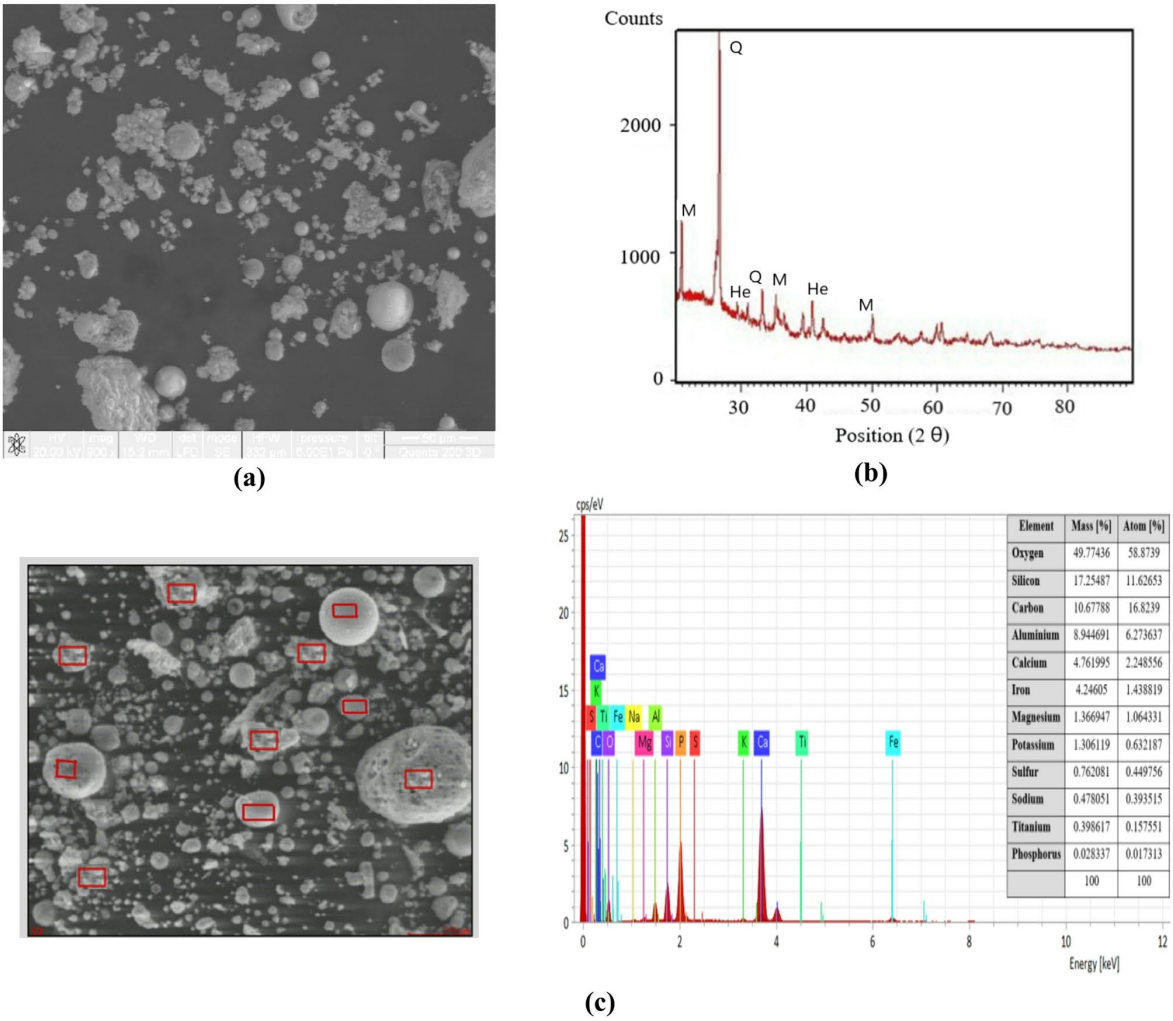
Romanian fly ash characterisation: **(a)** scanning electron microscopy analysis; **(b)** X-ray diffraction analysis; **(c)** energy-dispersive X-ray spectroscopy analysis.

**Fig. 3:**
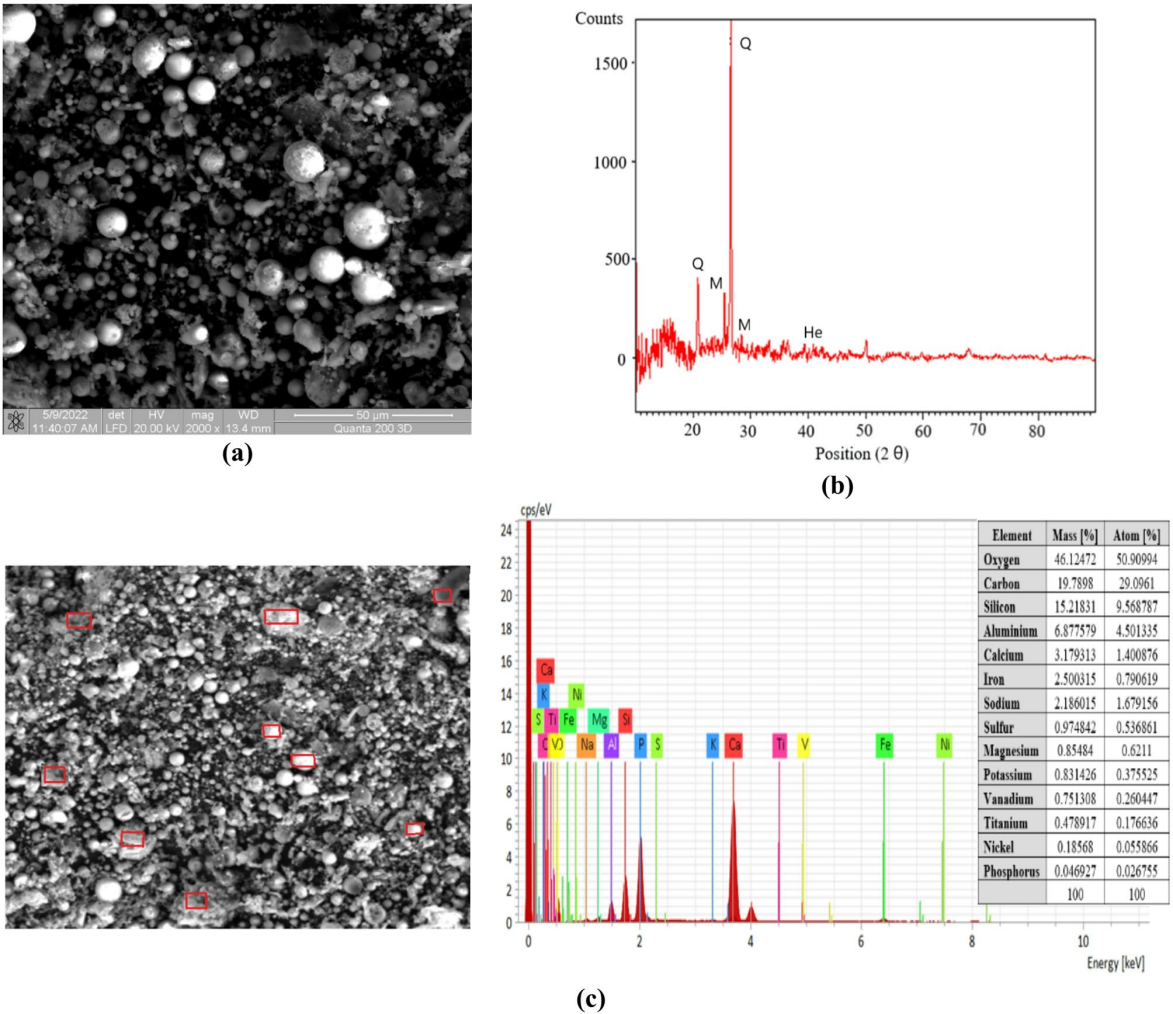
Canadian fly ash characterisation: **(a)** scanning electron microscopy analysis; **(b)** X-ray diffraction analysis; **(c)** energy-dispersive x-ray spectroscopy analysis.

The raw materials used for the synthesis of AIMs were subjected to a complete characterisation. Fly ash, resulting from electro-filters from power plants, is the most widely used source of aluminosilicate for obtaining AIMs. Because the annual production of fly ash is of billion tonnes, these by-products pose serious environmental concerns if not properly preserved and disposed^31^. The characterisations of Romanian fly ash (supplied by CET Iasi) and Canadian fly ash are presented in Figs. 2 and 3. The element in the highest concentration is silicon, followed by oxygen and aluminium, which provide the basis for the geopolymer structure^32^.

The morphology of fly ash particles is affected by factors such as the coal source, combustion conditions, and cooling rate. In this study, particle sizes ranged from below 1 μm up to 25 μm. Most particles are solid spheres, while irregularly shaped unburned carbon particles typically have a larger size distribution. Minerals and mineral aggregates often exhibited surface melting. The irregularly shaped particles represent unburnt carbon (Figs. 2(a) and 3(a)), a fact confirmed by elemental analysis. The elemental analysis of the fly ash proves that it contains Si, O, C, Al, Ca, Fe, K, V, Na, Mg and Ti (Figs. 2(c) and 3(c)), in accordance with the literature^33,34^.

Both fly ashes are primarily vitreous materials (as indicated by the halo observed for 2θ = 20–30°). They also contain several minor crystalline phases, including quartz (SiO_2_, JCPDS 05–0492), mullite (3Al_2_O_3_·2SiO_2_, JCPDS 15–0776), and magnetite (Fe_3_O_4_, JCPDS 19–0629). The XRD spectra in Figs. 2(b) and 3(b) show that the fly ashes contain quartz (Q), hematite (He), mullite (M), and a glassy phase. Peaks below 15 θ correspond to carbon and are observed to be more intense in the case of Romanian ash (Figs. 2(b) and 3(b)). The peak at the value of 25 θ corresponding to quartz is sharp and has the highest intensity. Based on the data presented in the elemental analysis, the analysed ashes fall into class F.

Romanian fly ash particles exhibit a spherical or irregular shape. The smooth surface texture of spherical particles typically makes them less reactive in the alkali activation process compared to irregularly shaped particles. Canadian fly ash has a globular and smooth surface morphology, which suggests a consistent and controlled combustion process. The crystallinity of the fly ash plays a critical role in the reactivity of the material when activated. The amorphous (non-crystalline) fraction of Romanian fly ash was lower compared to Canadian fly ash, reducing the overall reactivity of the material in the alkali activation process. EDX analysis demonstrated that both ashes contain the same elements, but differences in silicon and aluminium content of Canadian fly ash facilitate the formation of more stable phases like N-A-S-H (sodium–alumino–silicate–hydrate), which ensures that it exhibits superior mechanical properties over time. On the other hand, a negative influence was the content of unburnt carbon found in Romanian ash. The choice between Romanian and Canadian fly ash for AIMs depends on the desired properties, but the Canadian fly ash offers better long-term performance.

#### Blast furnace slag

The granulated blast furnace slag, is a by-product of the metallurgical industry, that can be used in building industry, due to its cementitious properties. It offers several benefits for mortar, including easy availability, chemical resistance, and excellent thermal properties. Combining slag with fly ash class F enhances the reactivity of alkali binders with a low calcium concentration.

**Fig. 4:**
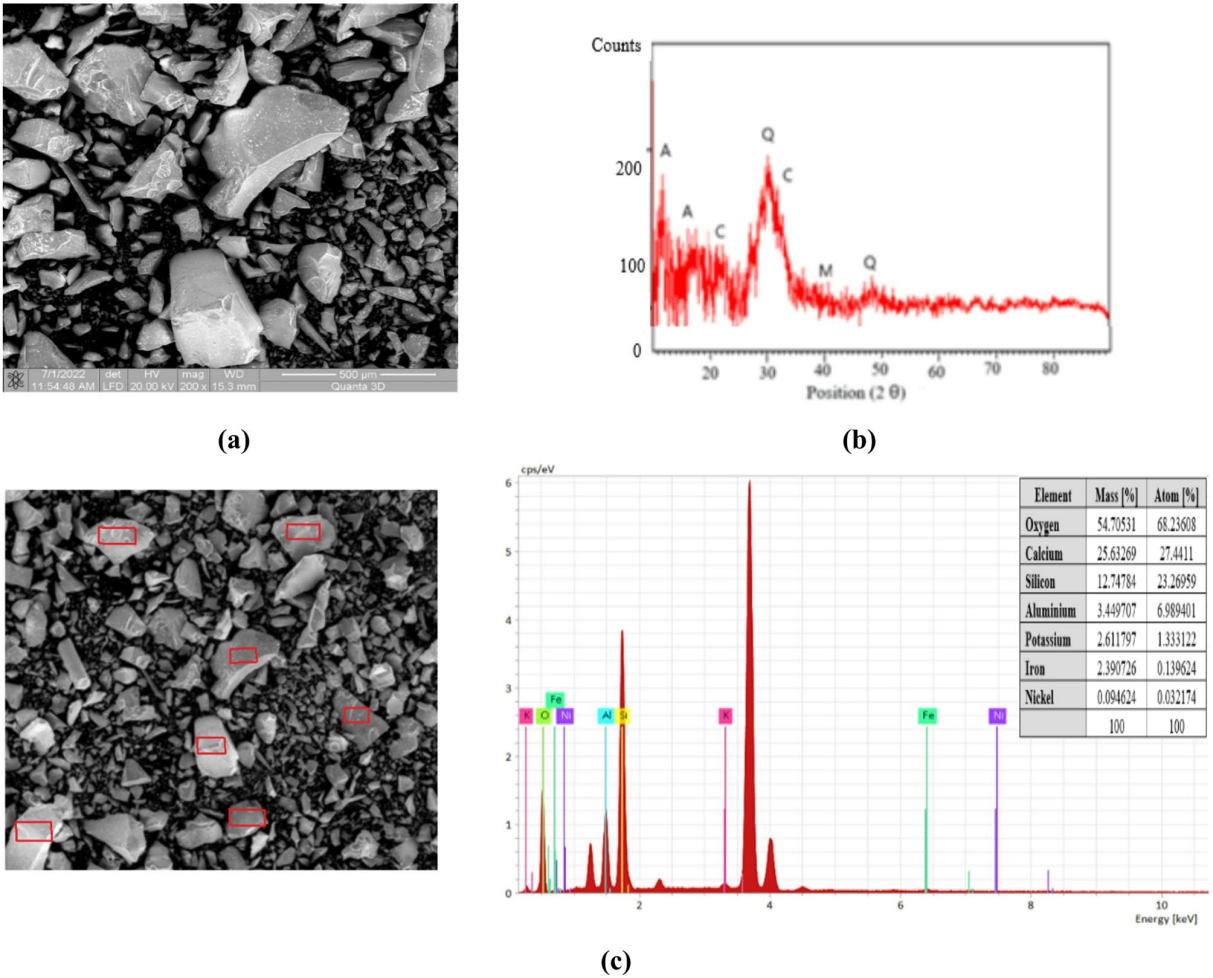
Granulated blast furnace slag characterisation: **(a)** scanning electron microscopy analysis; **(b)** X-ray diffraction analysis; **(c)** energy-dispersive x-ray spectroscopy analysis.

In order to be able to use granulated blast furnace slag to obtain AIMs, it first needs to be characterised; its characterisation is shown in Fig. 4.

The SEM image shown in Fig. 4(a) shows that the particles are predominantly angular in shape. The surface is uneven, and the appearance of a vitreous phase (resulting from the rapid cooling process of the molten iron slag) can be observed^35^.

The identification of the chemical elements in the slag was conducted using elemental chemical analysis, with the results displayed in Fig. 4(c). The composition (in mass and atomic percentages) reveals that the slag contains Ca, Si, O, Al, Fe, and traces of K and Ni.

Based on the elemental mass composition, the oxidic chemical composition was calculated and presented as follows: CaO, 38.136%; SiO_2_, 25.71%; Al_2_O_3_, 14.98%; Fe_2_O_3_, 7.35%; and K_2_O, 0.329%. The high calcium oxide content should be noted from the chemical characterisation. Also, the analysed slag has a high aluminium oxide content.

The XRD analysis shown in Fig. 4(b) reveals that the analysed slag has a slightly crystallised structure, which positively impacts the process of producing AIMs. The slag contains quartz (Q), mullite (M), akermanite (A) and calcite (C). The diffractogram pattern indicates the presence of calcium–aluminium–silicate compounds, as reported in the specialised literature^36^.

#### Silica fume

**Fig. 5:**
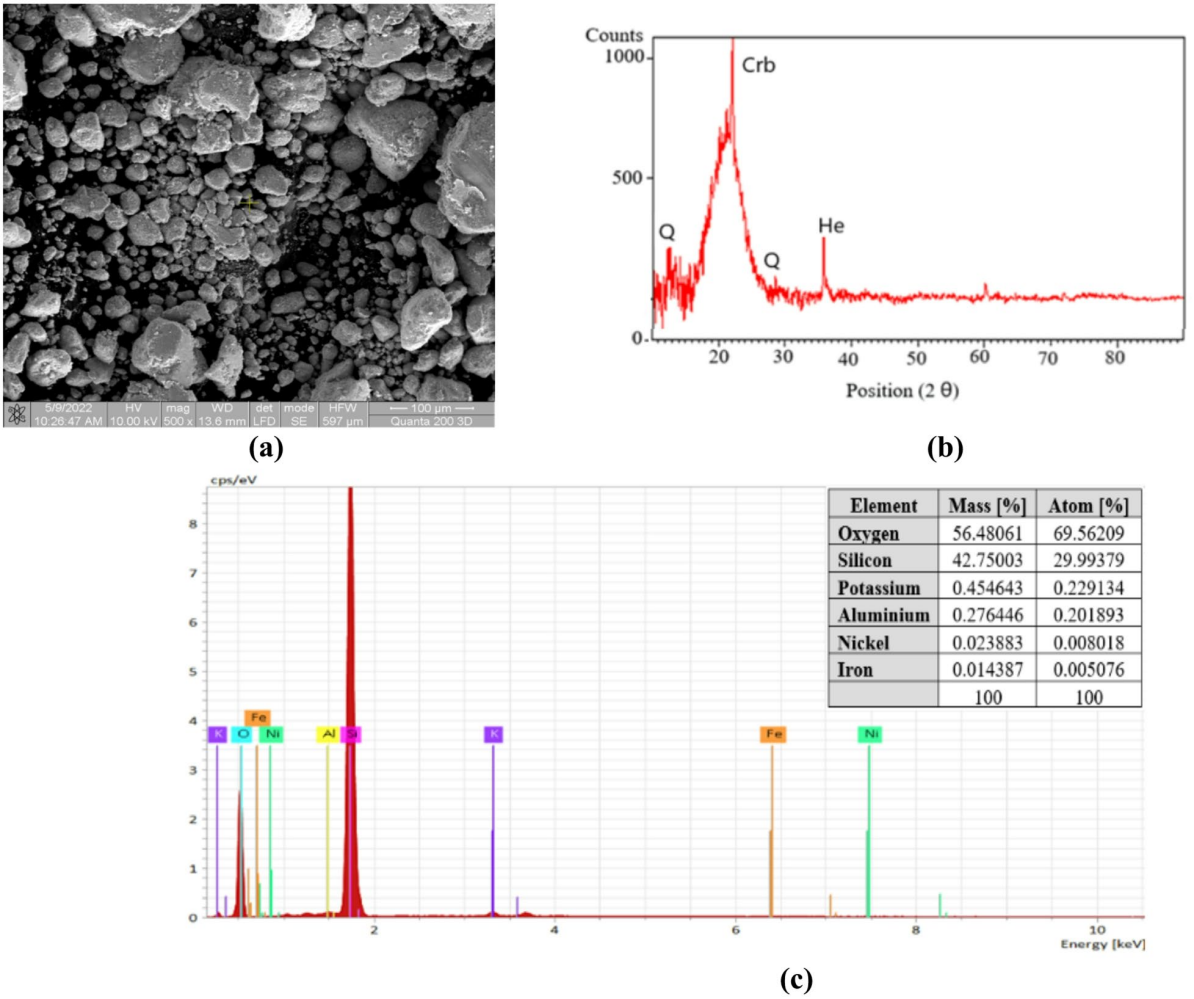
Silica fume characterisation: **(a)** scanning electron microscopy analysis; **(b)** X-ray diffraction analysis; **(c)** energy-dispersive x-ray spectroscopy analysis.

Silica fume, a by-product from manufacturing of ferrosilicon alloy, is an ultrafine powder. The silica fume consists of small particles of amorphous silica (SiO_2_), with particle sizes between 30 and 300 nm. The characterisation of silica fume is presented in Fig. 5.

Silica fume possesses a very high specific surface area and functions as a reactive pozzolan. It is typically used in smaller quantities than other pozzolanic materials. A larger amount of water is needed to hydrate the silica fume, so it is also necessary to use a superplasticiser or a water reducer. Figure 5(a) shows that the silica fume has predominantly spherical particles, but some irregular shapes are also present, with particles of different sizes, up to 50 μm. Most of the particles are of an irregular shape, and the rest are microparticles of spherical to irregular shape, the morphology being determined by the temperature used to obtain and cool the silica fume. Particle size and shape have a main influence over the properties of the formulated material.

Figure 5 (c) shows that the key elements in the silica fume are Si, O, Al, K and Fe, with traces of Ni. The oxide composition of the silica fume is as follows: SiO_2_, 95.54% (≥ 92); K_2_O, equiv. to 0.58% (≤ 1.0); Al_2_O_3_, 0.54%; and Fe_2_O_3_, 0.032%.

Silica fume mainly contains silicon oxide, but the production process also results in silicon carbide (the content must be less than 3%), free carbon (which determines the grey colour), and small amounts of impurities from the raw materials.

The chemical elements present in silica fume appear in crystalline form, as seen in the XRD diagram in Fig. 5(b). XRD analysis shows that the silica fume contains cristalobalite (Crb), quartz (Q) and hematite (He).

To obtain AIMs with the desired characteristics, the chemical components of the raw material used in its composition must be easily activated^36^.

### Characterisation of the AIMs

The AIMs in a first stage were subjected to characterisation of their compressive and flexural strengths. Two types of fly ash were used as the main source of aluminosilicate, differing in their chemical composition, particle size and the amount of unburnt carbon. The main way to design this material was to determine the compressive strength as well as the flexural strength.

### Mechanical properties of hardened mortar

Compressive strength is one of the key factors in determining the mechanical properties of a construction material. High values for the compressive strength can be obtained by the proportionality of the binder, sand, alkaline liquid and other components. Compressive strength was determined on cubic samples with dimensions 40 × 40 × 40 mm according to standard SR EN 12190:2002^37^. The flexural strength of a sample was determined by applying a three-point load, until breaking, on prismatic samples of hardened mortar. The prismatic samples have dimensions of 40 × 40 × 160 mm according to standard SR EN 1015-11:2020^38^.

**Fig. 6:**
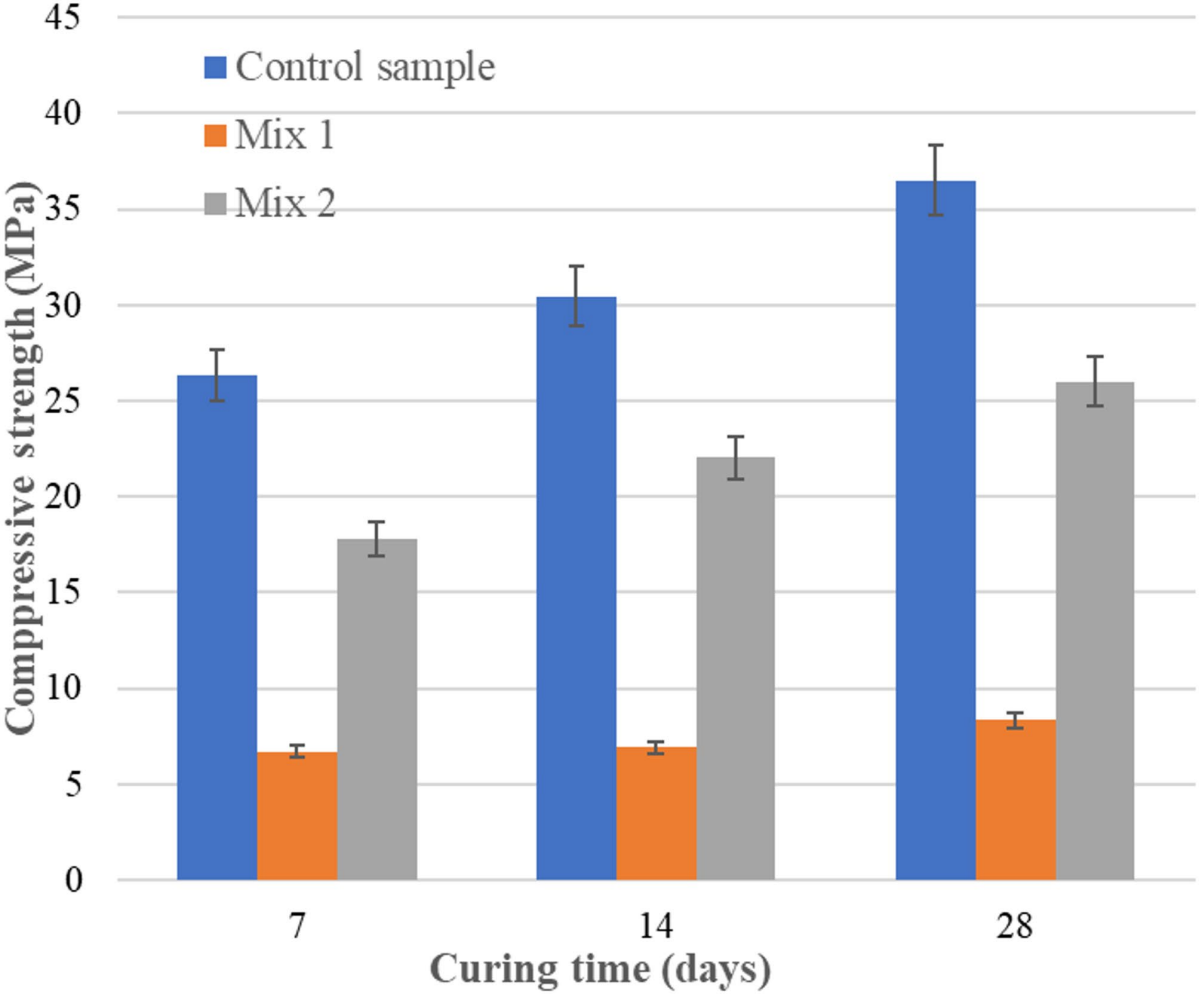
Compressive strengths (MPa).

**Fig. 7:**
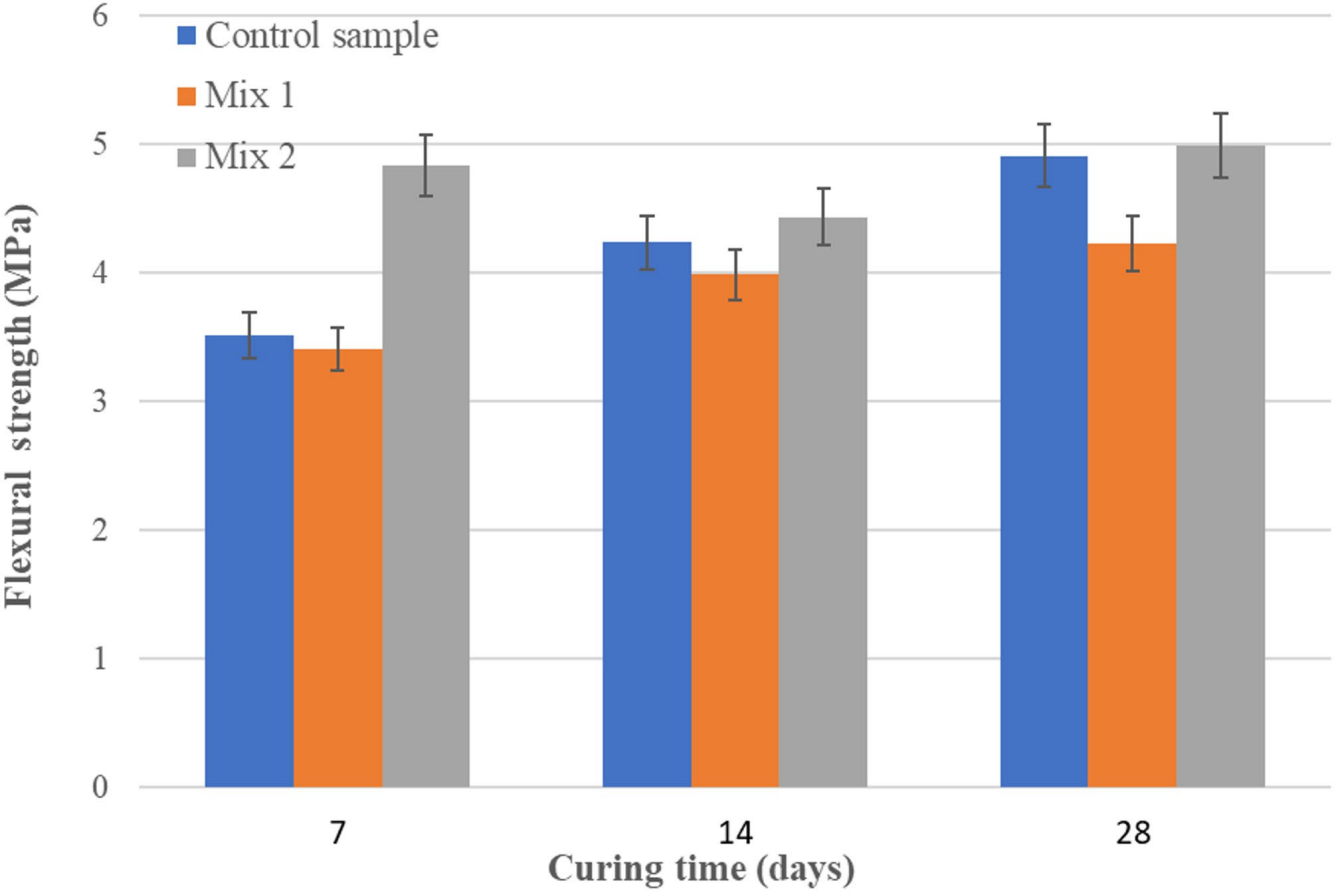
Flexural strengths (MPa).

In order to perform these tests, a 15/250 kN hydraulic press was used. The tests were performed in accordance with standard^38^(7, 14 and 28 days). Figures 6 and 7 present the values obtained from the compressive and flexural tests on Mix 1 and Mix 2 samples, each containing fly ash sourced from different locations. These tests were performed to establish the influence of ash sources. The values were compared with a control sample of mortars based on cement. The first set of values was obtained using Romanian ash, while values obtained with Canadian ash showed a very high increase. Comparatively with Romanian fly ash, in Mix 2, on the base of Canadian fly ash, the compressive strength values doubled. If the curing time increase from 7 to 28 days, compressive strength increased with 45% (Fig. 6). A smaller increase was observed in the case of flexural strength, only 15% from 7 to 28 days (Fig. 7). However, the obtained values are lower compared to the control sample. This observation led to the decision to use the Canadian ash to continue the experiment. The high carbon content of the Romanian ash led to materials with improper mechanical properties. As a result, the next experiments were conducted with Canadian ash (Mix 2), and furnace slag was used to improve the properties (Mix 3).

**Fig. 8:**
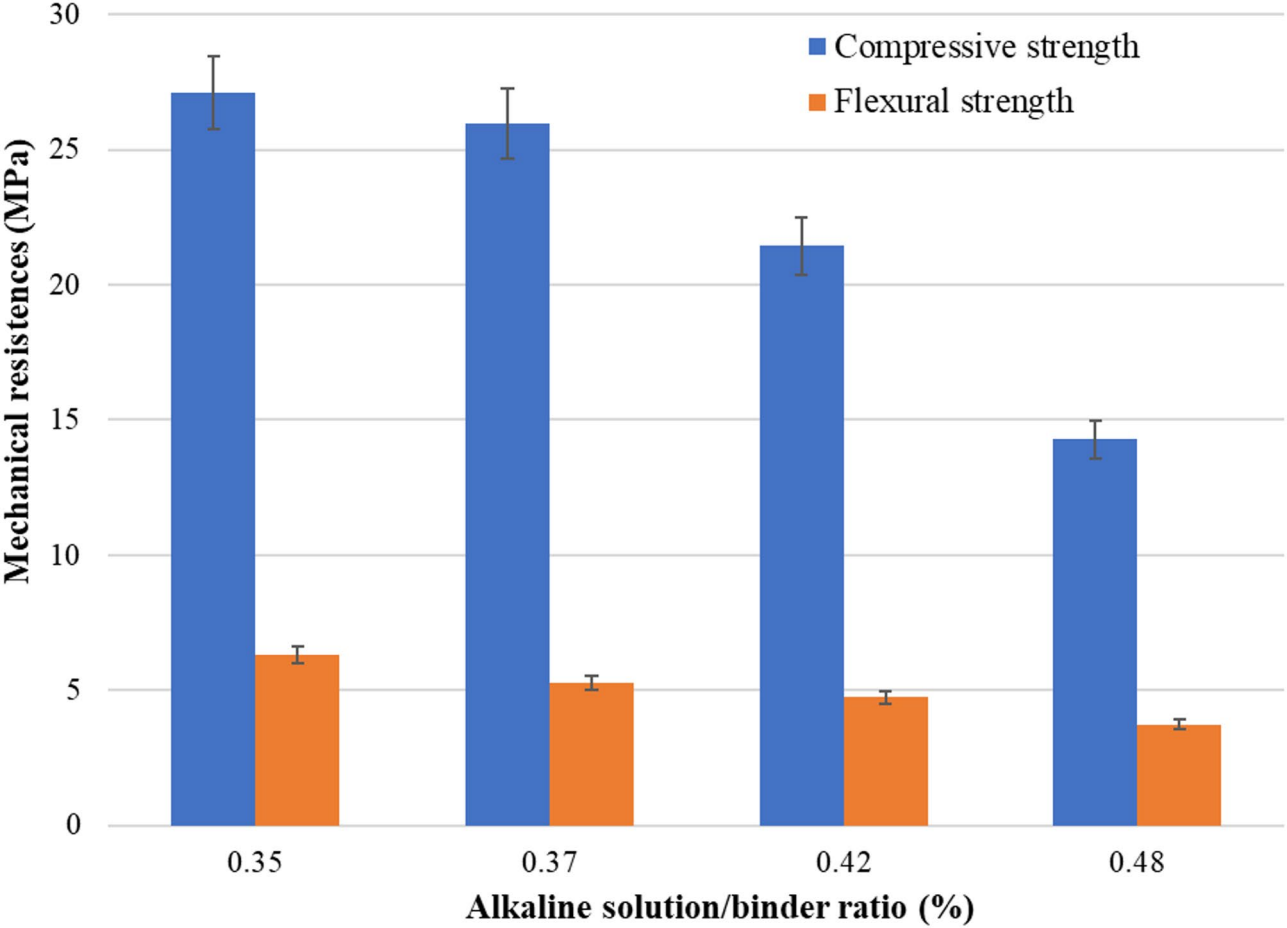
Variation of the mechanical strengths of Mix 2 as a function of alkaline solution/binder ratio.

The influence of alkaline solution/binder over mechanical properties was tested in Mix 2 (Fig. 8). The results show how a high alkaline solution/binder ratio negatively affects the mechanical strength. An alkaline solution/binder ratio of 0.48 weakens the mechanical strength, especially compressive strength, because it disrupts the material’s internal structure. Materials can have voids, increased porosity, and weaker bonds between particles. Reducing the ratio from 0.48 to 0.35 leads to an increase in compressive strength by more than 40%.

**Fig. 9:**
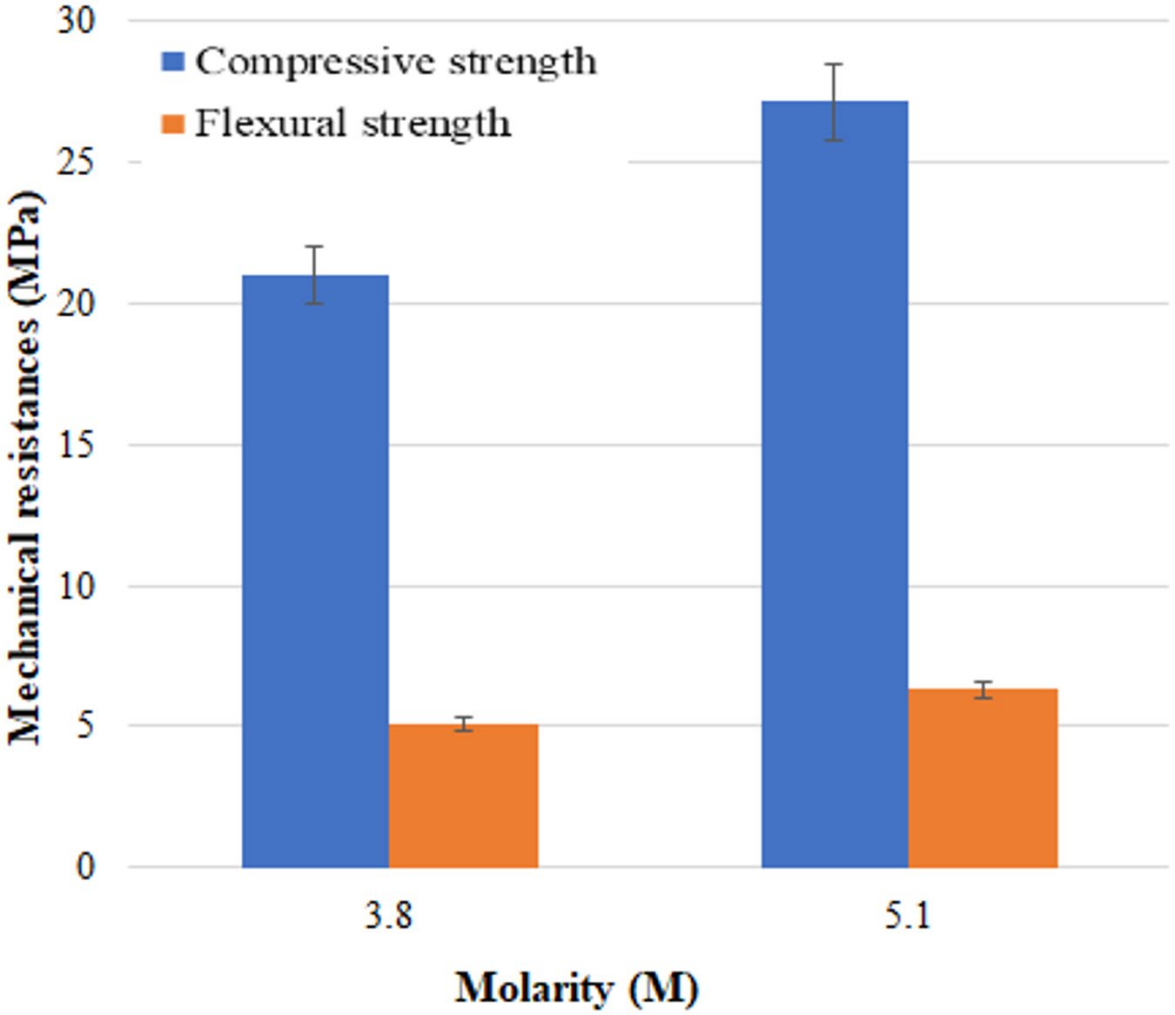
Variation of the mechanical strengths of Mix 2 as a function of potassium hydroxide concentration.

The influence of KOH molarity on the mechanical properties of binder Mix 2 is presented in Fig. 9. The maximum value of 5.1 M was chosen for cost reasons and to prevent excesses that could cause pollution. These factors were considered while also ensuring compliance with the recommended resistance classes for mortars. A concentration of 5.1 M leads to higher strengths (27.12 compressive strength and 6.3 MPa for flexural strength) compared to a solution of 3.8 M (20.9 compressive strength and 5.08 MPa for flexural strength). One reason would be that the silicon and aluminium in the fly ash are dissolved much faster in a more concentrated KOH solution.

A higher KOH concentration leads to a better activation reaction and, implicitly, a denser aluminosilicate gel. Through the alkali activation of some industrial wastes, we obtained new inorganic materials with characteristics similar to those of materials based on OPC. The chemical composition of the AIM resembles that of zeolites, although its microstructure is amorphous. The polymerisation process involves a rapid chemical reaction under alkaline conditions on the raw materials, leading to the formation of a three-dimensional polymer chain structure composed of Si-O-Al-O bonds.

These AIMs show mechanical characteristics comparable to classical cement-based materials. The technology of inorganic materials in the hardened state is directly correlated to the mineralogical composition of the pozzolan used. Slight variations in these secondary products have marked effects on the properties of the AIMs obtained. The amount of calcium in the raw material has a strong influence on determining the reaction path and the physical characteristics of the desired product. Before activation, it is necessary to carry out a microanalysis of the source material to identify the minerals present, as well as their quantity.

By adding a certain percentage of furnace slag and undensified silica fume, the mechanical resistance can be improved. Furthermore, by reducing the porosity of the resulting material, the value of the compressive strength increases. The very fine size of undensified silica fume particles greatly improves chemical resistance by forming a very dense structure that is difficult to penetrate by chemicals such as weak acids, concentrated salt solutions (chlorines, sulphates) and highly caustic solutions.

Silica fume greatly reduces water permeability and minimises the penetration of chloride ions, which cause corrosion of reinforced steel. The material reduces segregation due to the addition of fine particles, which increase the cohesion of the final product.

**Fig. 10:**
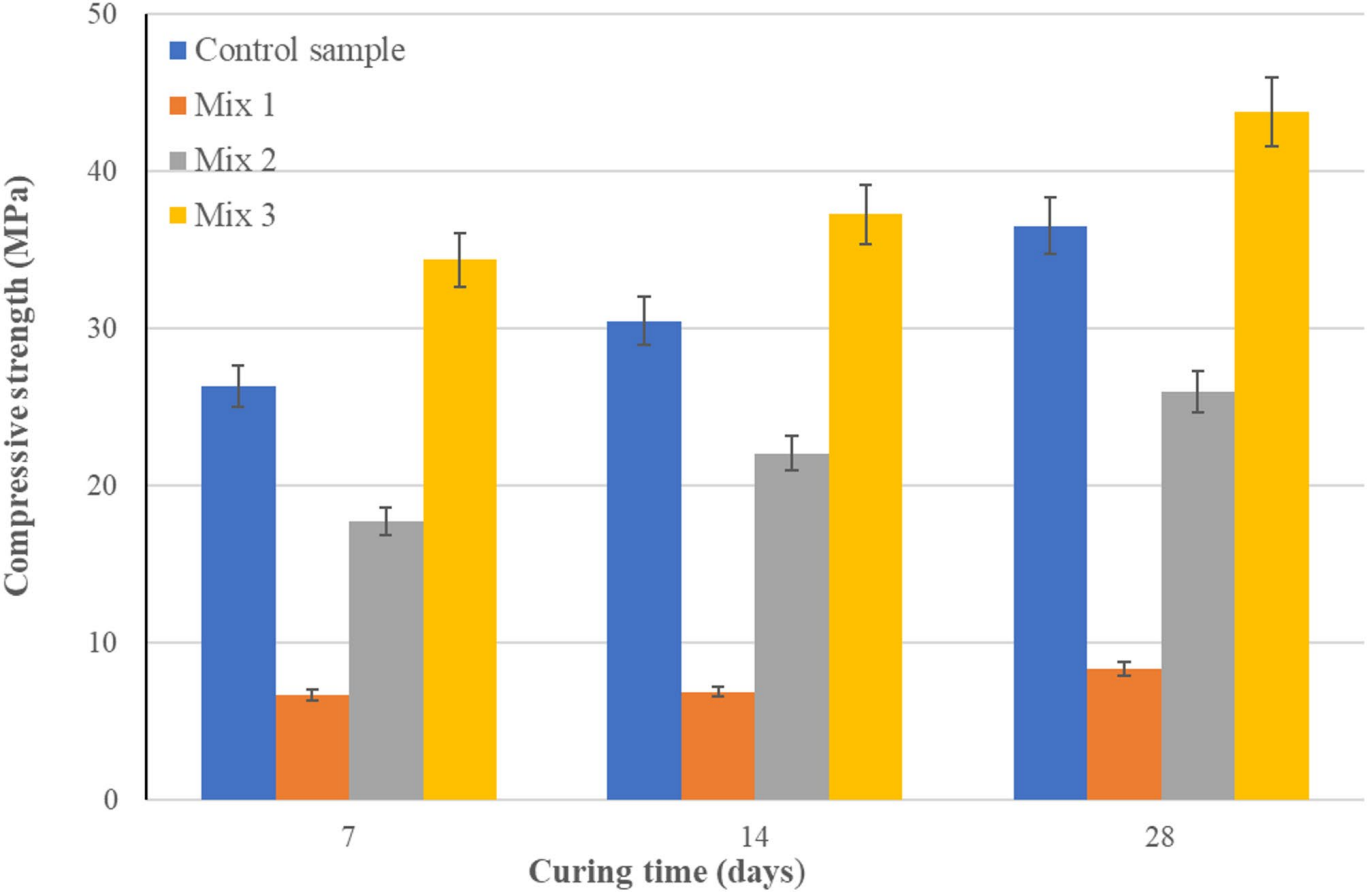
Compressive strengths of synthesised alkaline inorganic materials.

Figure 10 presents the mechanical strengths of the AIMs, which uses, as an aluminosilicate source, fly ash of Canadian origin with a certain percentage of furnace slag and silica fume. In the case of the material with furnace slag and silica fume incorporated, a uniform increase in compressive and flexural strength can be observed, and the increase is gradual. The blast furnace slag in the formulation leads to compressive and flexural strengths at 28 days of 43.75 MPa and 7.41 MPa, respectively, in accordance with the literature^39^.

The medium values obtained for compressive strength are shown in Fig. 10. The Canadian ash resulted in a compressive strength of 25.99 MPa, compared to 8.34 MPa for the Romanian ash (Mix 2 vs. Mix 1). Mix 3 exhibited the highest compressive strength, reaching approximately 37 MPa at 14 days and 43 MPa at 28 days, highlighting the positive effect of using blast furnace slag and Canadian fly ash. In contrast, Mix 1 showed the lowest strength at all curing times, suggesting a lower reactivity of Romanian fly ash, explained by physico-chemical properties of it. Mix 2 demonstrated intermediate performance, indicating that while Canadian fly ash alone performs better than Romanian fly ash, the addition of slag nearly doubles strength. The chemical composition of slag has a positive effect over activation processes. The control sample showed moderate strength development, underlining the superior performance of the proposed AIMs mixtures.

#### Microstructural properties

**Fig. 11:**
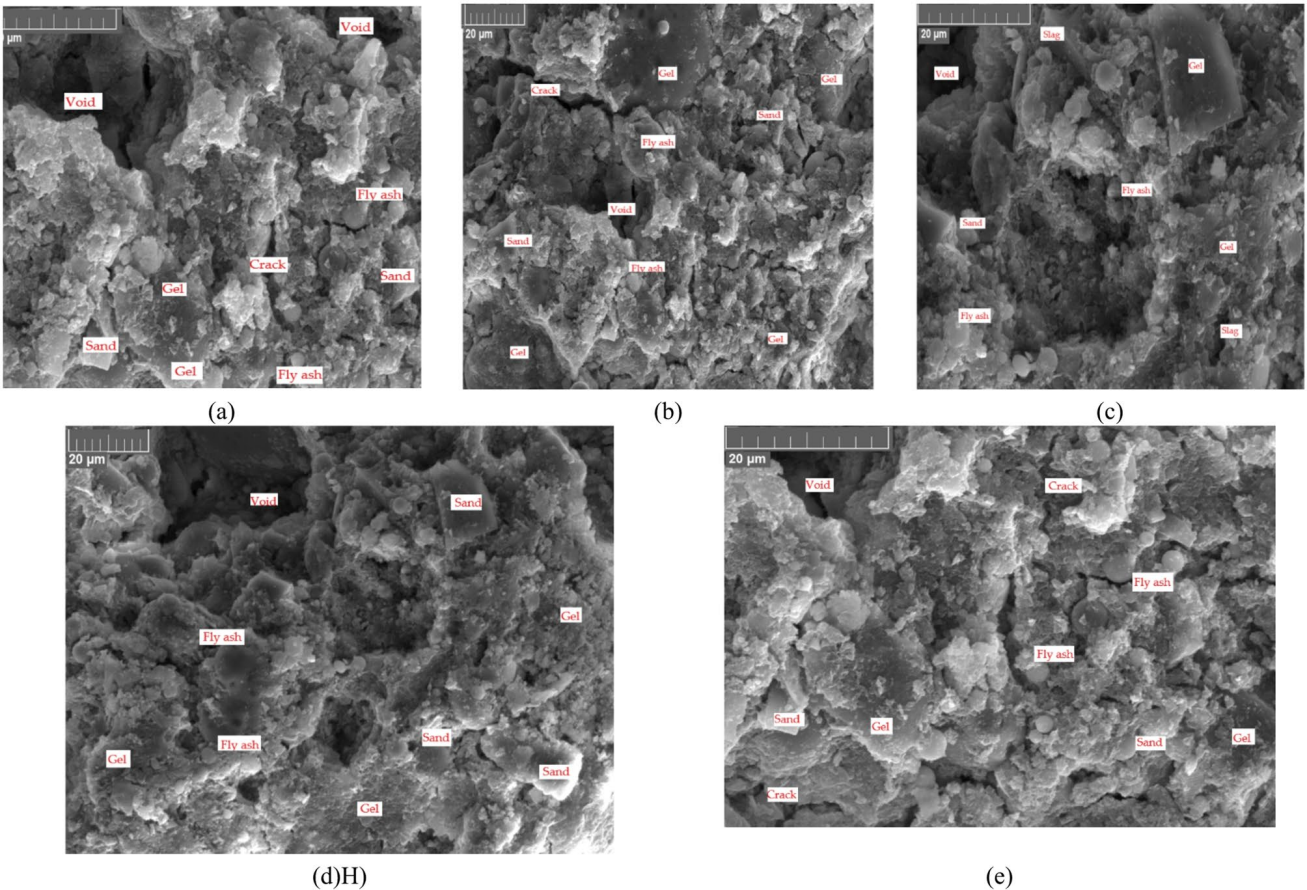
Microstructural properties of the alkali-activated materials. (**a**) – Mix 1, (**b**) – Mix 2, (**c**) – Mix 3, (**d**) – Mix 2 activated with 5.1 M potassium hydroxide, (KOH), (**e**) – Mix 2 activated with 3.8 M KOH.

The SEM images of the AIM samples are presented in Fig. 11. The microstructure of AIMs changes during the alkali activation process, forming a complex network of reaction products that influence properties. Morphologically, the samples presented a uniform structure, function of type of ash, sodium silicate/KOH ratio and molarity. AIMs tend to have a more homogeneous microstructure than OPC mortars. The activation process involves breaking down and restructuring the microstructure of the alumino–silicate material, leading to a more uniform distribution of the binder phase. The obtained AIMs exhibit smaller pore sizes. The microstructure typically consists of a combination of gel phases and unreacted precursor particles, with the gel phase forming a dense network around the unreacted grains (Fig. 11). In SEM images, gel phases are one of the most important structures to observe. The gel presents a dense, amorphous structure, which is indicative of its role as the binding matrix in the material. The gel is predominantly observed in Mix 2 and Mix 3, formed by the polymerisation of silicate and aluminate species, fills the space between the sand and unreacted particles, resulting in a compact structure. In addition to the gel phase, unreacted particles, such as fly ash or slag, can also be seen in SEM images. Fly ash particles typically appear as smooth, spherical particles, while slag particles appear rough and angular. The presence of these unreacted particles suggests that the activation process was incomplete. The pores visible in the SEM images are often a result of capillary action during the activation process or due to the excess water used during mixing, particularly in the case of the 3.8 M KOH solution. Another critical issue observed in SEM images is the presence of microcracks, which often appear between the gel phase and the unreacted particles. These cracks indicate poor bonding between the phases and can affect the structural integrity and mechanical properties of the material.

The formation of the aluminosilicate gel took place because the degree of alteration of the ash was high, but the presence of cracks, untransformed ash particles and voids in the material can also be observed. In Mixes 1 and 2, small cracking is observed; however, the addition of furnace slag (Mix 3) improves the morphology. This improvement is demonstrated by the superior mechanical strengths of Mix 3. By analysing the influence of activator concentration, it can be concluded that decreasing the concentration negatively affects cracking (as shown in Figs. 11(d) and 11(e)).

#### Thermogravimetric analysis

**Fig. 12:**
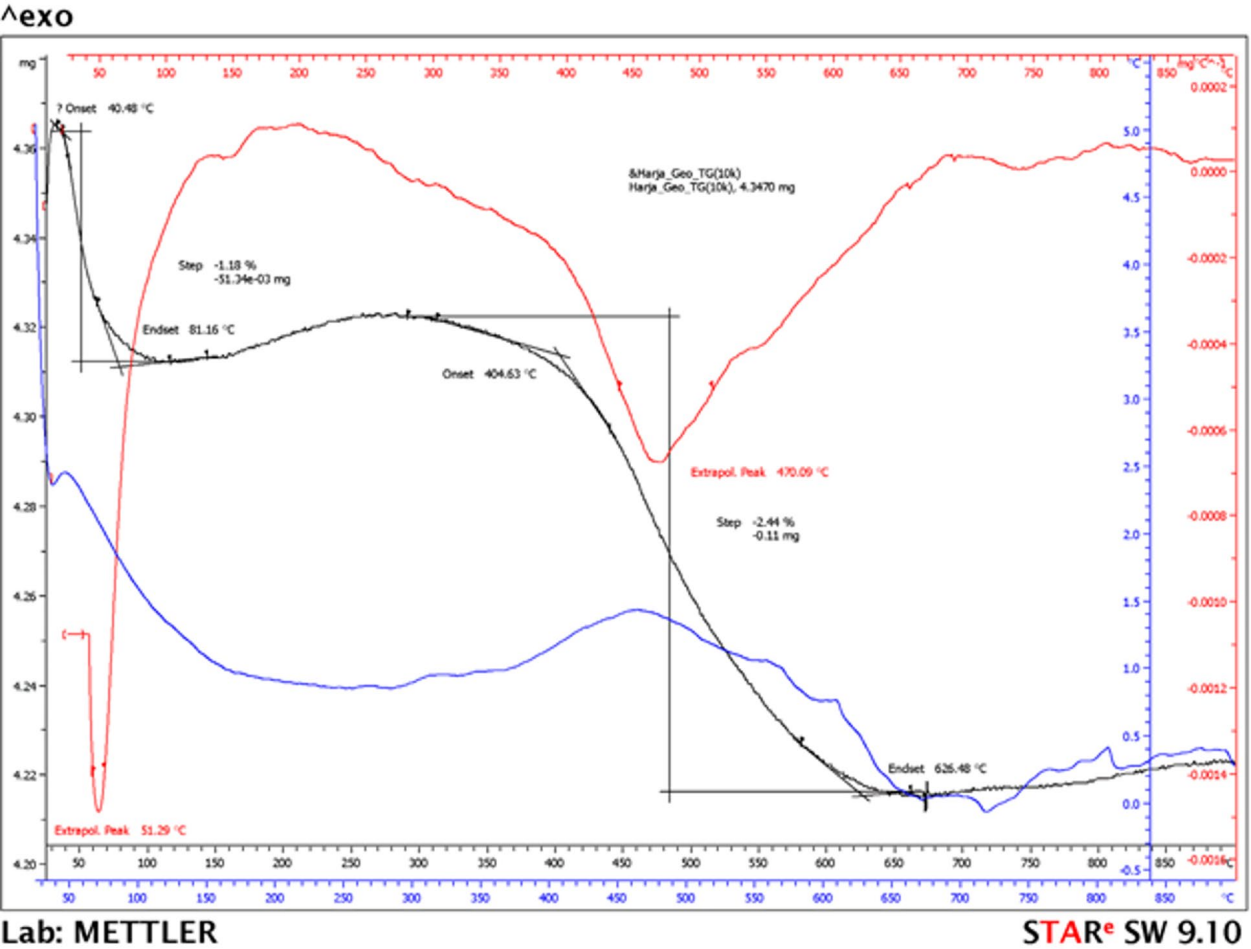
Thermogravimetric analysis of Mix 2 in air.

In the current study, the two-stage mass loss observed in AIM mortars is consistent with the literature^40–42^. The first stage, at lower temperatures, suggests dehydration, while the second mass loss indicates the loss of water from crystallohydrates. The results obtained for the Mix 2 sample are shown in Fig. 12. On analysing the data in Fig. 12, it is found that the mass loss occurs through heating, which starts at 40℃ and ends at 626℃, and takes place in two stages. The highest mass loss of 2.44% occurs at maximum speed in the second stage, at 470℃. For Mix 2 the total mass loss is 3.62%.

Regarding Mix 1 and Mix 3, the termogravimetric curves are similar with Mix 2, but the total mass loss was 3.59% for Mix 1 and up to 2% for Mix 3. This lower value of mass loss at higher temperatures indicates a more robust gel with better resistance to thermal degradation.

### Carbon footprint of AIMs

AIMs are often lauded for their potential to reduce CO₂ emissions when compared to OPC. The emissions reduction is mainly due to lower calcination temperatures and the use of industrial by-products which would otherwise contribute to waste disposal problems. The impacts of the production^43^, processing and transportation of the raw material substantially contribute to greenhouse gas emissions^44,45^.

**Fig. 13:**
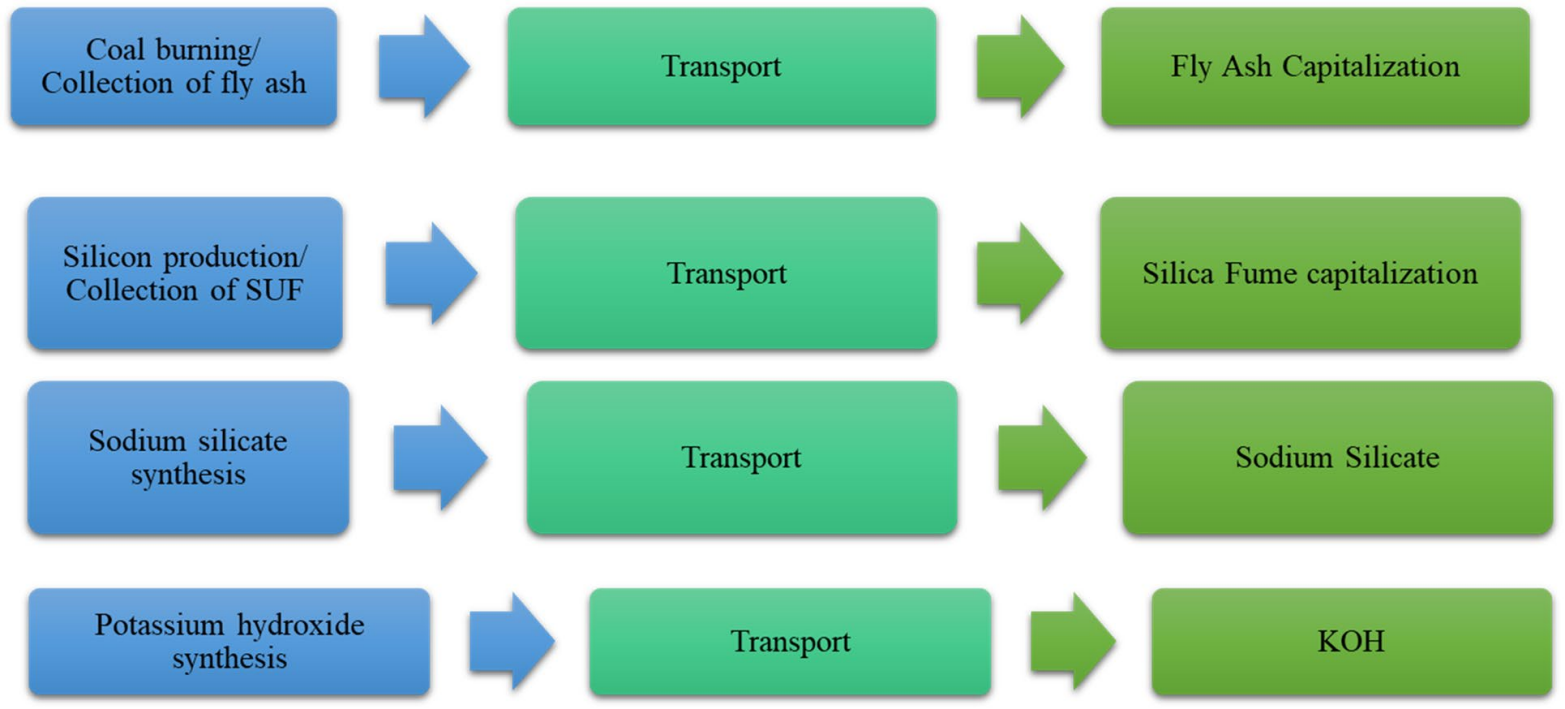
Production stages of the raw material used to obtain the alkaline inorganic materials.

**Fig. 14:**
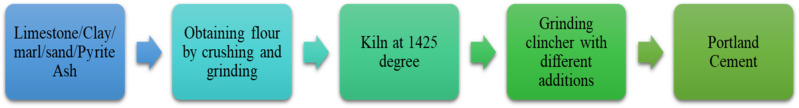
Production stages for ordinary Portland cement.

The production stages of the raw material used to obtain AIMs compared to materials based on OPC are illustrated in Figs. 13 and 14, highlighting the influence of the extraction of the raw material and its production. Energy (fuel and electricity consumption), CO_2_ emissions and cost are three important factors that are considered to be the focal argument for the use of AIMs^46^.

Transport of raw materials at all stages through to production of the alkali material is of vital importance because the values of costs and emissions can be greatly affected by the distance as well as the mode of transport^47^. AIMs typically involve the use of by-products from various locations, and the transportation of these raw materials can account for a large proportion of the total CO₂ emissions. The transportation considerations that have a direct impact on CO₂ emissions include the distance from the production site to the manufacturing plant, emission factors applied based on the type of vehicle used and the distance covered, and the mode of transportation. Road transportation tends to have a higher CO₂ emission factor per ton-kilometre compared to rail or sea transport, while the type of fuel used in transportation is another important consideration. The emissions factor (gCO₂ per km per ton of material transported) depends on the fuel efficiency of the vehicle and the type of fuel used.

The curing conditions of AIMs—such as temperature and time—can affect both the CO₂ emissions during production and the material’s long-term environmental impact. Many studies have suggested that lower curing temperatures or the use of ambient curing conditions can reduce the carbon footprint of AIMs. For instance, Qu et al.^48^ found that low-temperature curing methods could further reduce CO₂ emissions.

Equation (1) can be used to calculate the CO_2_ emissions released by the OPC-based materials and the AIMs^49^:

1 $$\:Emission=\sum\:_{i=1}^{n}{m}_{i}({d}_{i}{e}_{i\:}+{p}_{i})$$

where m_i_ = mass of component i; d_i_ = distance transported; e_i_ = emission factor (function of transportation mode); and p_i_ = emission factor for raw materials.

Table 2 presents the carbon emission factors for raw materials included in the composition of the cement-based and AIM mortar^50,51^.

**Table 2:** Carbon emission factors for the raw materials (kg CO_2_-eq per 1 kg).

Components	*p*_i_
Fly ash	5.26 10^− 3^
Silica fume	3.13 10^− 4^
Ground blast furnace slag (GBFS)	1.69 10^− 2^
Metakaolin	9.25 10^− 2^
Superplasticiser	7.49 10^− 1^
Fine sand, aggregates	2.4 10^− 3^
Free water	1.55 10^− 4^
KOH	1.94
Sodium silicate (3.3 WR, 37% solids)	1.96
Sodium metasilicate pentahydrate	1.24

On the basis of the block diagram in Fig. 14 using Eq. (1), without transport, the amount of CO_2_ released in the case of the AIM obtained in this study was calculated; the resulting value was 220 kg CO_2_/m^3^, which is in accordance with the literature^52,53^. The impact of transportation on CO_2_ emissions from the proposed materials is in the range of 20–25% for the AIM produced using Canadian fly ash, which indicates that the much greater distances achieved by the raw materials directly affected the amount of CO_2_ released.

The literature contains various comparative studies on the amount of CO_2_ released for materials based on OPC and alkali-activated waste. This comparative study is presented in Table 3.

**Table 3:** Amount of CO_2_ released by the cement- and waste-based materials.

Materials	kg CO_2_-eq/m^3^	References
Concrete (340 kg OPC/m^3^)	316	^54^
Concrete (440 kg OPC + 120 kg FA/m^3^)	237	^55^
Concrete (360 kg OPC/m^3^)	341	^56^
Concrete (234 kg OPC/+ 87 kg FA/m^3^)	233	^57^
Concrete (288 kg OPC/+ 44 reactive waste/m^3^)	279	^57^
Geopolymer concrete	286.8	^55^
Geopolymer concrete	288.7–291	^57^
Geopolymer concrete	244	^54^
Geopolymer concrete	366.85	^58^
Mortar (378 kg OPC + 162 kg GBFS)	405	^59^
Mortar (270 kg OPC + 270 kg GBFS)	300	^59^
Mix 2	219.03	This study
Mix 3	222.7	This study

It can be concluded that the amount of CO_2_ released is in the range of 316–340 kg/m^3^ for OPC-based materials and 180–280 kg/m^3^ for AIMs. In terms of percentages, there is the potential to reduce greenhouse gas emissions^60^. The AIM’s emissions decreased with about 30% when compared to OPC concrete and 45% when compared with OPC mortar (378 kg OPC + 162 kg GBFS/m^3^), confirming that AIMs are lower-carbon mortars.

By comparing the AIMs with cement mortars, Table 3 shows that AIMs align with the findings from studies such as Zhu et al.^61^, which show that using higher amounts of industrial by-products can lead to lower CO₂ emissions. Faridmehr^62^ highlight that AIMs using by-products lead to lower CO₂ emissions due to the avoidance of the high-temperature processes involved in the production of OPC. The use of by-products in AIMs is known to reduce the need for limestone and the energy-intensive production of clinker, which is the primary source of CO₂ emissions in OPC production.

Despite the promising results, this study presents certain limitations. The CO₂ emissions did not include transportation impacts, which could affect the overall CO₂ assessment. In addition, the experiments were conducted at laboratory scale and limited to a 28-day curing period, while long-term durability and large-scale performance were not investigated. Furthermore, the variability of industrial by-products and the restricted range of activator molarities may influence the general applicability of the findings.

Future research will explore other industrial by-products, varied activator molarities from 2 M to 5 M, and long-term durability to enhance performance and applicability. The laboratory results may be validated at industrial scale, to evaluate the feasibility and performance of AIM mortars.

## Conclusions

When evaluating the performance of AIMs in comparison to OPC, it is essential to explore the environmental benefits and mechanical advantages.

The main conclusions of this study are as follows:

1. Fly ashes, silica fume, and furnace slag were chemically and mineralogically characterised to assess their potential for the utilization of these wastes. The studied wastes improved the mechanical properties of the mortar obtained.
2. For mortars without furnace slag, the use of a 5.1 M KOH activator resulted in superior mechanical performance, achieving compressive and flexural strengths of 27.12 MPa and 6.3 MPa, respectively, compared to 20.9 MPa and 5.08 MPa obtained with a 3.8 M solution.
3. The AIM mortar containing furnace slag activated at 20 °C with 5.1 M KOH solution, and a 40% sodium silicate solution, had a higher compressive strength of 43.75 MPa and flexural strength of 7.4 MPa after 28 days, in accordance with standard requirements.
4. The utilization of the studied industrial by-products helps prevent their accumulation, while markedly reducing the consumption of natural raw materials, such as limestone and clay.
5. The AIM mortar exhibited a 45% reduction in CO₂ emissions compared with OPC mortar incorporating GBFS.
6. With a maximum mass loss of only 3.62% at 850 °C, obtained AIMs exhibit thermal stability.
7. Materials obtained in this paper represent a promising alternative to OPC mortars.
8. Experimental results demonstrated that AIMs were obtained under the investigated conditions using 5.1 M KOH solutions with an activation process that occurred at room temperature.

## Data Availability

The data presented in this study are available on request from the corresponding authors.
